# Honokiol is a FOXM1 antagonist

**DOI:** 10.1038/s41419-017-0156-7

**Published:** 2018-01-24

**Authors:** Marianna Halasi, Ben Hitchinson, Binal N. Shah, Renáta Váraljai, Irum Khan, Elizaveta V. Benevolenskaya, Vadim Gaponenko, Jack L. Arbiser, Andrei L. Gartel

**Affiliations:** 1 Department of Medicine, University of Illinois, Chicago, IL USA; 2 Department of Biochemistry and Molecular Genetics, University of Illinois, Chicago, IL USA; 3 Department of Dermatology, Emory University School of Medicine, Atlanta Veterans Administration Medical Center, Atlanta, Georgia USA

## Abstract

Honokiol is a natural product and an emerging drug for a wide variety of malignancies, including hematopoietic malignancies, sarcomas, and common epithelial tumors. The broad range of activity of honokiol against numerous malignancies with diverse genetic backgrounds suggests that honokiol is inhibiting an activity that is common to multiple malignancies. Oncogenic transcription factor FOXM1 is one of the most overexpressed oncoproteins in human cancer. Here we found that honokiol inhibits FOXM1-mediated transcription and FOXM1 protein expression. More importantly, we found that honokiol’s inhibitory effect on FOXM1 is a result of binding of honokiol to FOXM1. This binding is specific to honokiol, a dimerized allylphenol, and was not observed in compounds that either were monomeric allylphenols or un-substituted dihydroxy phenols. This indicates that both substitution and dimerization of allylphenols are required for physical interaction with FOXM1. We thus demonstrate a novel and specific mechanism for FOXM1 inhibition by honokiol, which partially may explain its anticancer activity in cancer cells.

## Introduction

Forkhead family member Forkhead Box M1 (FOXM1) is ubiquitously expressed in a wide range of human cancers and it contributes to several different aspects of oncogenesis^1^. Because of its key role in cancer development, FOXM1 emerged as an important and relevant candidate of therapeutic intervention^2^. However, some might argue that being a transcription factor FOXM1 cannot be easily targeted by conventional drug development strategies and it might represent an “undrugable” target. Previously, we found that proteasome inhibitors target FOXM1^3^ and recently we determined the mechanism for the suppression of FOXM1**:** proteasome inhibitors stabilize HSP70, which binds to FOXM1 and inhibits the activity of FOXM1 as a transcription factor^4^. We demonstrated that after binding to FOXM1, HSP70 inhibits the DNA-binding of FOXM1 and its transcriptional activity. Because of the FOXM1 auto-regulation loop HSP70-mediated inhibition of FOXM1 transcriptional activity also leads to the suppression of its protein expression^4,5^.

Honokiol is a small molecular weight dihydroxylated biphenyl isolated from the genus Magnolia^6,7^. Previous studies have shown activity against common epithelial tumors (breast, lung, pancreatic, prostate)^8–11^, hematologic malignancies (chronic lymphocytic leukemia, myeloma)^12,13^, and sarcomas (angiosarcoma, osteosarcoma)^14,15^. Honokiol has antitumor activity as a single agent, but has synergy with additional chemotherapeutic agents, consistent with its effect on NFkB activation^9^. While honokiol inhibits NFkB transcriptional activity, it is not known to directly bind NFkB subunits^16^. Most recently, honokiol has been shown to promote mitochondrial normalization by inducing the mitochondrial enzyme Sirt3^17^.

In the current study, we discovered that honokiol targets oncogenic transcription factor FOXM1 by a mechanism different from proteasome inhibitors. Honokiol exerts its inhibitory activity on FOXM1 via binding to FOXM1 in a specific manner, while closely related allylphenols and unsubstituted hydroxybiphenyls have no effect. We demonstrate that honokiol after binding to FOXM1 inhibits FOXM1 transcriptional activity and because of FOXM1 auto-regulation loop it also decreases FOXM1 mRNA and protein expression. Overall, we found that honokiol is a novel antagonist of FOXM1 and inhibition of FOXM1 may play a critical role in its anticancer activity.

## Results and discussion

### Honokiol binds FOXM1 and inhibits transactivation by FOXM1

**Fig. 1:**
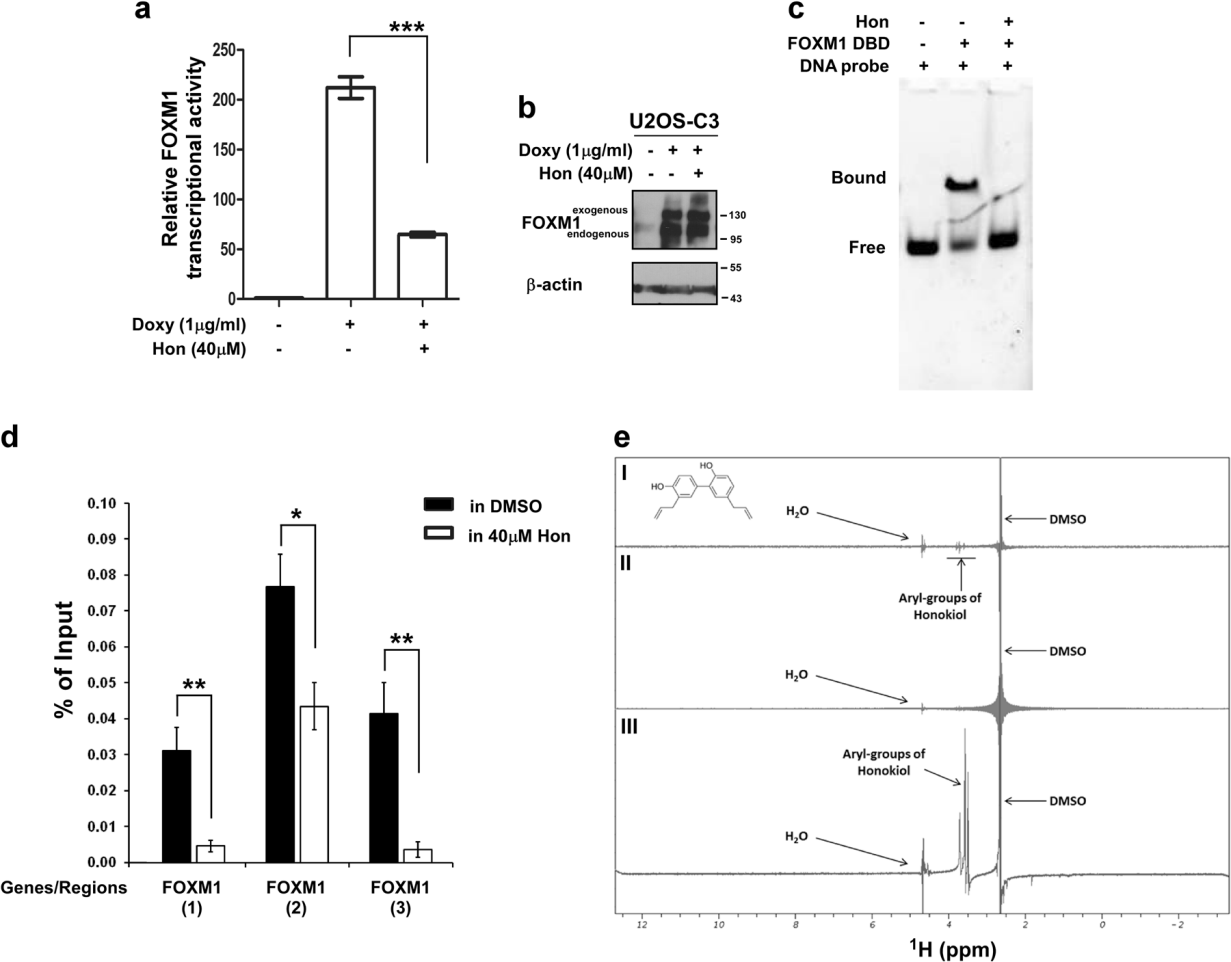
Honokiol inhibits FOXM1 transactivation via binding **a** C3-luc cells were induced with doxycycline and treated with honokiol for 24 h. The luciferase activity was determined by using the Luciferase Assay System (Promega). Graph shows quantification as fold induction of firefly luciferase activity compared to control cells, mean ± SD of a representative triplicate experiment. **b** The C3 cell line was treated with doxycycline and honokiol in the indicated concentrations for 24 h. Cells were collected and immunoblotting was performed with a FOXM1 specific antibody. β-actin was used as the loading control. **c** Representative EMSA image shows the inhibitory effect of honokiol on the formation of the FOXM1 DBD protein–DNA complex. **d** The C3 cell line was treated with doxycycline and honokiol as indicated for 24 h. Then, cells were processed for the ChIP experiments, as described in “Materials and methods”. Graph shows mean ± SEM of two independent ChIP experiments. **e** Saturation transfer difference (STD) NMR spectra to assess the binding of honokiol to FOXM1: (I) 2 mM of honokiol alone, (II) 150 ng of recombinant FOXM1 alone, (III) 2 mM honokiol with 150 ng of recombinant FOXM1. The chemical structure of honokiol is illustrated. STD signals arising from the aryl groups in honokiol are annotated, and signals from vehicle (DMSO) and water are labeled

To evaluate the effects of honokiol on FOXM1 transcriptional activity, we utilized the U2OS-derived C3-luc cell line^18^ with stable expression of the doxycycline-inducible FOXM1-GFP fusion protein and the 6× FOXM1b-TATA-luciferase reporter plasmid. Following addition of doxycycline to the culture media, FOXM1-related firefly luciferase activity increased several fold (Fig. 1a). Similarly to bona fide proteasome inhibitors^3^, honokiol significantly inhibited FOXM1-dependent transcription (Fig. 1a), suggesting that honokiol is able to interfere with the transcriptional activity of FOXM1 even in the presence of excess amount of exogenous FOXM1 (Fig. 1b).

Electrophoretic mobility shift assays (EMSA) were performed to examine the effect of honokiol on FOXM1 DNA-binding in vitro. The FOXM1-binding site DNA duplex oligonucleotide^19^ was incubated with recombinant FOXM1 DNA-binding domain (DBD) protein in the presence or absence of honokiol for 1 h at room temperature. The FOXM1 DBD protein–DNA complexes were resolved by electrophoresis and the DNA probe bands were visualized by fluorescence imaging. Honokiol significantly reduced the DNA-binding of recombinant FOXM1 DBD protein (Fig. 1c).

Next, we investigated the relevance of the inhibition of FOXM1 transcriptional activity by honokiol in human cancer cells. Chromatin Immunoprecipitation (ChIP) experiments were carried out to determine whether honokiol interferes with FOXM1 DNA-binding in cancer cells. FOXM1 expression was increased by doxycycline addition to the C3 cell line with stable expression of the doxycycline-inducible FOXM1-GFP fusion protein^20^. Simultaneously, the cells were also treated with honokiol for 24 h. After treatment the cells were processed for ChIP. The ability of FOXM1 to bind DNA in the presence of honokiol was tested on its own promoter region. As a result of these quantitative ChIP assays we found that honokiol strongly reduces FOXM1 binding to its regulatory elements (Fig. 1d), while the levels of exogenous FOXM1 was not affected (Fig. 1b). These data suggest that honokiol inhibits FOXM1 activity as a transcription factor by hindering its binding to DNA.

To investigate whether the effects of honokiol were a consequence of binding to FOXM1, we performed saturation transfer difference (STD) nuclear magnetic resonance (NMR) experiments with recombinant, full length FOXM1 in the presence of honokiol. This method allows for the investigation of small molecule binding to high molecular weight proteins, such as FOXM1. Initial experiments indicated that at 2 mM concentrations, honokiol aggregated, generating an STD signal without the addition of the protein (Fig. 1e-I). Upon the addition of 150 ng of FOXM1, we observed an increase in the signal intensity between 3 and 4 ppm indicating the binding of the aryl-groups of honokiol to FOXM1 (Fig. 1e-III).

### Honokiol suppresses FOXM1 expression, but not as a proteasome inhibitor

**Fig. 2:**
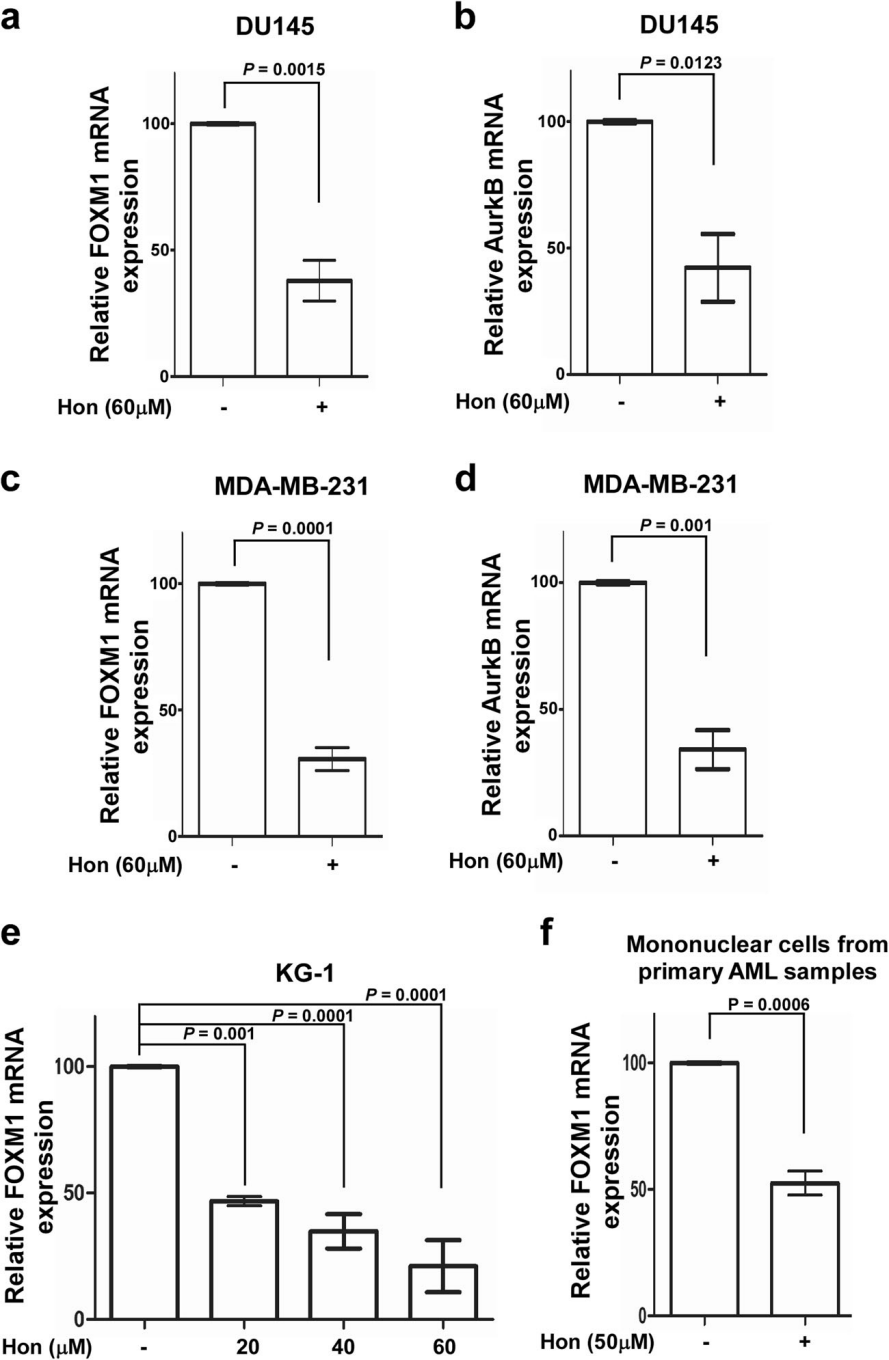
FOXM1 expression is also downregulated after honokiol treatment **a**–**f** DU145 prostate, MDA-MB-231 breast cancer cells, KG-1 leukemia and mononuclear cells from primary AML samples were collected for RNA extraction after the indicated treatments. Quantitative real time PCR was carried out with FOXM1^5^ and AurkB^30^ primers. Graph shows quantification as percentage of mRNA expression levels in treated cells compared to control cells, mean ± SEM of three independent experiments

**Fig. 3:**
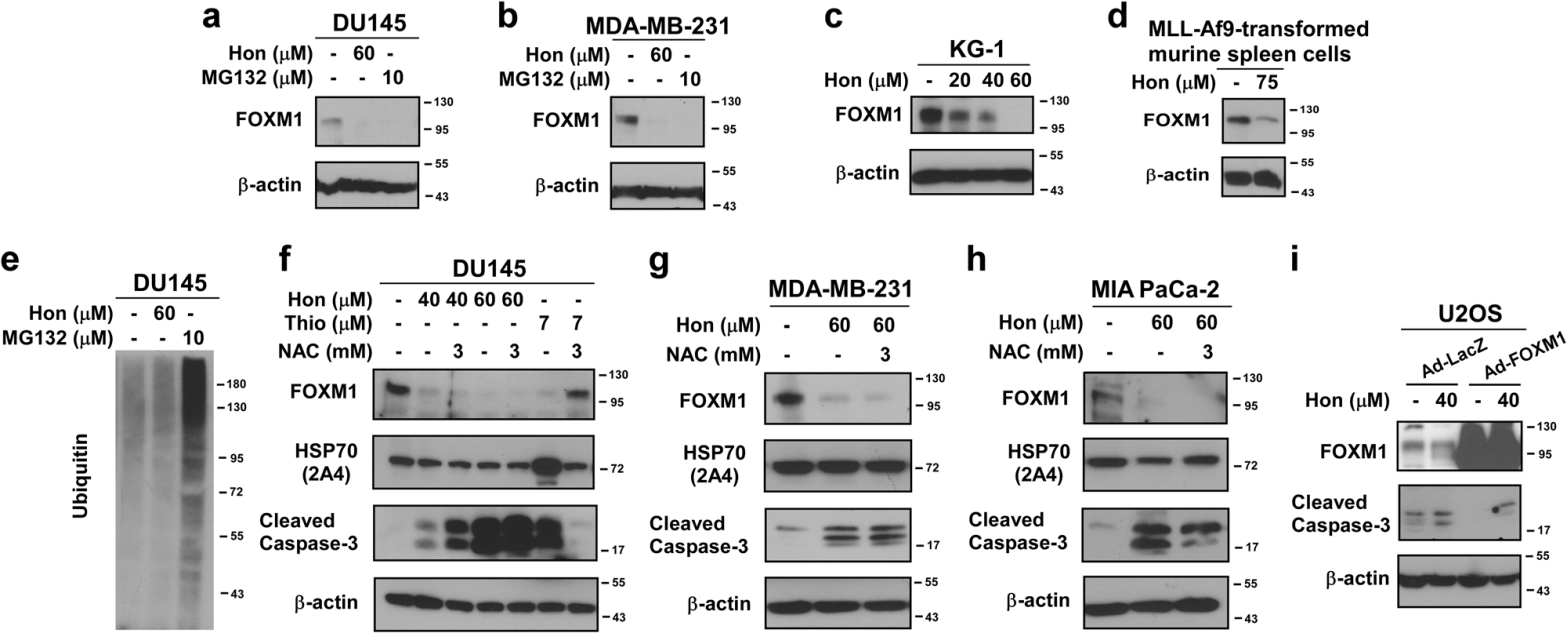
Honokiol does not exhibit proteasome inhibitory activity **a**–**d** DU145, MDA-MB-231 cancer cells, KG-1 leukemia cells, and MLL-AF9 oncogene transformed murine spleen cells were treated with honokiol or proteasome inhibitor MG132 as indicated. 24 h after treatment cells were collected and immunoblotting was carried out with antibodies against FOXM1 and β-actin as the loading control. **e** DU145 prostate cancer cells were treated with the indicated concentrations of honokiol or MG132. 24 h after treatment cells were collected and immunoblotting was performed for ubiquitin. **f**–**h** DU145 prostate, MDA-MB-231 breast and MIA PaCa-2 pancreatic cancer cells were treated with the indicated concentrations of honokiol or FOXM1/proteasome inhibitor thiostrepton in the absence or presence of NAC. Cells were collected 24 h after treatment and the protein level of FOXM1, HSP70 and cleaved caspase-3 was assessed by immunoblotting. β-actin was used as the loading control. **i** U2OS osteosarcoma cells were transduced with LacZ or FOXM1 carrying adenoviral particles. 24 h after transduction cells were treated with 40 µM honokiol for an additional 24 h. Immunoblotting was performed with FOXM1 and cleaved caspase-3 specific antibodies. β-actin was used as the loading control

FOXM1 regulates its own transcription via a positive feedback loop^5,21^, therefore inhibition of FOXM1 transcriptional activity also results in its decreased mRNA and protein levels^18,22^. We previously identified proteasome inhibitors as FOXM1 inhibitors^3^ and we also found that they downregulate FOXM1 via the upregulation of HSP70^4^. Because treatment with honokiol led to the suppression of FOXM1 transcriptional activity (Fig. 1a) we also tested how it affects its mRNA and protein expression. Using quantitative real-time PCR, we found that the mRNA levels of FOXM1 itself and FOXM1 transcriptional target Aurora B kinase were reduced more than 50% after honokiol treatment for 24 h in DU145 prostate, MDA-MB-231 breast cancer cells, KG-1 leukemia cells and in mononuclear cells from the blood of newly diagnosed AML patients (*n* = 3) (Fig. 2). Furthermore, breast (MDA-MB-231), prostate (DU145) cancer cells, leukemia (KG-1), and MLL-AF9 oncogene transformed murine splenocytes were treated with honokiol or bona fide proteasome inhibitor MG132. Western blot analysis showed that honokiol represses FOXM1 protein levels as efficiently as proteasome inhibitors (Fig. 3a–d).

FOXM1 suppression, along with formation of ubiquitin conjugates or stabilization of short-life proteins are hallmarks of proteasome inhibition^23^. Interestingly though, compared to treatment with proteasome inhibitors MG132 or thiostrepton, formation of ubiquitin conjugates (Fig. 3e) and stabilization of HSP70 were not detectable after honokiol treatment (Fig. 3f-h). Furthermore, NAC, which is an inhibitor of proteasome inhibitors^24^, was not able to reverse suppression of FOXM1 or apoptosis mediated by honokiol treatment in contrast to proteasome inhibitor thiostrepton (Fig. 3f–h). All these data provide further evidence that honokiol suppresses FOXM1 through a novel mechanism distinct from proteasome inhibition.

Because suppression of FOXM1 by honokiol in human cancer cell lines correlated with cell death (Fig. 3f–h), we also tested whether overexpression of FOXM1 protects against honokiol-induced apoptosis. Transient FOXM1 overexpression in U2OS osteosarcoma cells was accomplished by the transduction of adenoviral particles, and then followed by honokiol treatment for 24 h. We found that FOXM1-overexpressing cells were less sensitive to apoptosis after honokiol treatment, as detected by cleaved caspase-3 (Fig. 3i). Taken together, honokiol suppresses FOXM1 and induces apoptosis, but overexpression of FOXM1 confers resistance to honokiol-mediated apoptosis, suggesting that suppression of FOXM1 by honokiol is partly responsible for honokiol-induced apoptosis.

### Structural analogs of honokiol do not bind and do not suppress FOXM1

**Fig. 4:**
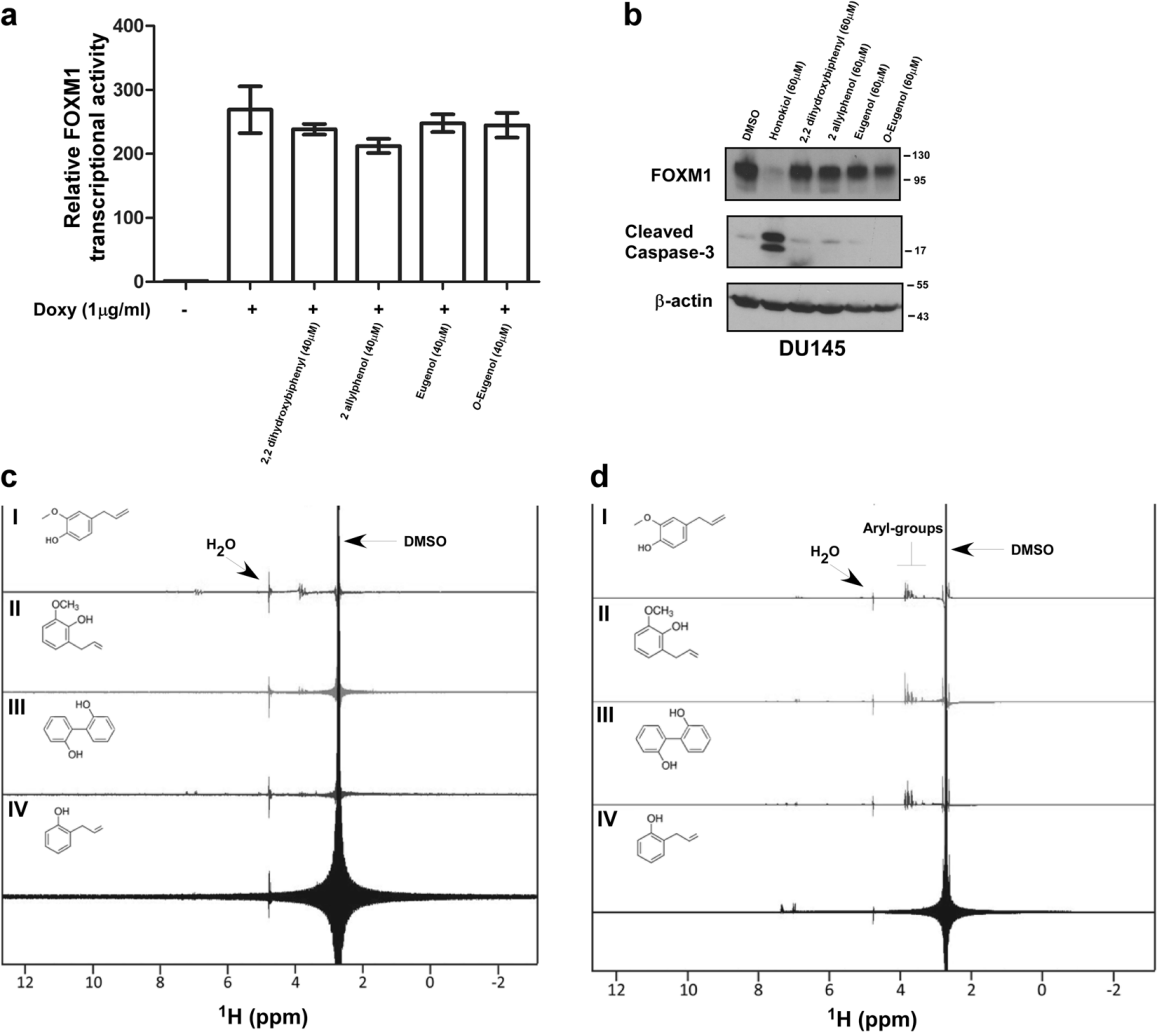
Analogs of honokiol do not bind or inhibit FOXM1 **a** C3-luc cells were induced with doxycycline and treated with the indicated analogs of honokiol at the concentration of 40 µM for 24 h. The luciferase activity was determined by using the Luciferase Assay System (Promega). Graph shows quantification as fold induction of firefly luciferase activity compared to control cells, mean ± SD of a representative triplicate experiment. **b** DU145 prostate cancer cells were treated as indicated. Following treatment cells were collected and immunoblotting was performed for FOXM1, cleaved caspase-3 and β-actin as the loading control. **c** STD NMR spectra of 2 mM of: (I) 2 mM eugenol, (II) 2 mM *O*-eugenol, (III) 2 mM 2-allylphenol and (IV) 2 mM 2,2 dihydroxybiphenyl. The chemical structure of each small molecule is illustrated. Signals from vehicle (DMSO) and water are labeled. **d** STD NMR spectra of 150 ng of recombinant FOXM1 plus: (I) 2 mM eugenol, (II) 2 mM O-eugenol, (III) 2 mM 2,2 dihydroxybiphenyl and (IV) 2 mM 2-allylphenol. The chemical structure of each small molecule is illustrated. STD signals arising from the aryl groups are annotated and signals from vehicle (DMSO) and water are labeled

In order to gain information for the structural requirements of honokiol’s inhibitory effect on FOXM1, we utilized various structural analogs of honokiol. Honokiol is a dimerized allylphenol, and we examined the effect of monomeric allylphenols, as well as unsubstituted dihydroxybiphenyls^6^. First, we looked at the transcriptional activity of FOXM1 in the presence and absence of the analogs utilizing the previously described C3-luc cell line (^18^, Fig. 1a). Unlike honokiol (Fig. 1a), treatment with the analogs did not inhibit FOXM1 transcriptional activity (Fig. 4a). Also, in contrast to honokiol, FOXM1 protein expression in different cancer cell lines was not affected by the honokiol analogs as detected by western blotting (Fig. 4b). Furthermore, the analogs did not induce apoptosis at the same concentration as honokiol did (Fig. 4b).

In addition, the analogs of honokiol that bear structural resemblance to honokiol, such as eugenol, O-eugenol, 2,2 dihydroxybiphenyl and 2-allylphenol did not interfere with FOXM1 DNA-binding in EMSA assays (data not shown) and showed little or no binding to FOXM1 in NMR experiments (Fig. 4c and d). This suggests that the presence of the two aryl-groups and their cis-orientation in the honokiol molecule are required for its binding to FOXM1 (Fig. 1e). Altogether, these data suggest that the effect of honokiol on FOXM1 has strict structural requirements, as neither the monomeric allylphenols, nor the unsubstituted dihydroxybiphenyls had inhibitory activity on FOXM1.

In summary, we characterized the effect of honokiol on oncogenic transcription factor FOXM1 in this study. Because FOXM1 is so centrally implicated in oncogenesis, in recent years it has become a prominent potential target of anticancer drug development^1,2^. However, no enzymatic function has been attributed to FOXM1, and therefore compounds that bind FOXM1 and inhibit its transcriptional activation are potentially useful. Honokiol is a small molecule that has demonstrated significant preclinical activity against multiple cancers with widely differing genetic backgrounds, including tumors with oncogenic Ras mutations^25^. We identified honokiol as a novel FOXM1 inhibitor. Similarly to HSP70, induced by proteasome inhibitors^3,4,18^, honokiol binds to FOXM1 and hinders its transcriptional activity (Fig. 1a). As a result of FOXM1 auto-regulation honokiol also inhibits FOXM1 mRNA and protein expression (Figs. 2 and 3). Honokiol does not demonstrate proteasome inhibitory activity based on accumulation of ubiquitin conjugates or stabilization of HSP70. In addition, NAC that has been shown to counteract proteasome inhibitors^24^ does not interfere with its function with respect to down-regulation of FOXM1 or induction of apoptosis (Fig. 3). Honokiol negatively regulates FOXM1 via binding to FOXM1 as demonstrated by NMR experiments (Fig. 1e). The binding of honokiol to FOXM1 is highly specific, because allylphenol monomers such as eugenol, and unsubstituted dihydroxybiphenyls do not bind to FOXM1 (Fig. 4). Our data suggest that the anticancer activity of honokiol is at least in part linked to the suppression of FOXM1. Because honokiol is a FOXM1 antagonist and it exhibits anticancer activity in a wide range of human cancers, honokiol deserves additional evaluation as an antitumor agent. FOXM1 was recently linked to worst outcomes in human cancers^26^, further confirming the importance of this transcription factor in cancer development. Therefore, FOXM1 inhibitors in the future may play a critical role in the treatment of cancer patients. This study may contribute to the development of more specific FOXM1 inhibitors.

## Methods

### Cell culture and chemical compounds

DU145 prostate, MIA PaCa-2 pancreatic cancer cell lines (ATCC), U2OS osteosarcoma and osteosarcoma-derived C3^20^ and C3-luc cells^18^ were grown in DMEM medium (Cellgro). MDA-MB-231 (ATCC) breast cancer cell line was grown in RPMI medium (Cellgro) and KG-1 (ATCC) leukemia cells in IMDM medium (GIBCO). The media were supplemented with 10% fetal bovine serum (Atlanta Biologicals) and 1% penicillin-streptomycin (GIBCO). Murine Leukemia Cells: Following 5-FU treatment to enrich for hematopoietic progenitor cells, primary murine bone marrow cells were transduced with retrovirus containing pMIG-FLAG-MLL-AF9. The pMIG-FLAG-MLL-AF9 was a gift from Daisuke Nakada (Addgene plasmid # 71443)^27^. Cells were subsequently transplanted into lethally irradiated syngeneic recipients. The spleens of the recipient mice were harvested after 3 months and their GFP positivity was quantified with flow cytometry and found to be 80%. Cells were cultured in RPMI with 10% FBS in the presence of IL3, IL6 and SCF (Peprotech). All the cells were maintained at 37 °C in 5% CO_2_. MG132 (EMD Millipore), thiostrepton (Sigma), honokiol, 2,2 dihydroxybiphenyl, 2 allylphenol, eugenol, and *O*-eugenol were dissolved in dimethyl sulfoxide (DMSO) (Fisher Scientific), N-acetyl-l-cysteine (NAC) (Sigma) in deionized water, and doxycycline (LKT Laboratories) in phosphate buffered saline (PBS). FOXM1 (Human) recombinant protein was purchased from Abnova and used for the NMR experiments.

### AML patient samples

Peripheral blood mononuclear cells from three patients with untreated AML were obtained from the University of Illinois Hematology Cell Bank (IRB protocol # 2015-0487). Cells were thawed and plated in liquid culture using StemSpan SFEM medium (Stemcell Technologies) serum-free medium supplemented by recombinant human cytokines (Flt3L, SCF, IL-3, IL-6, and TPO).

### Immunoblot analysis

Treated cells were collected and lysed by using IP buffer (20 mM HEPES, 1% Triton X-100, 150 mM NaCl, 1 mM EDTA, 1 mM EGTA, 100 mM NaF, 10 mM Na_4_P_2_O_7_, 1 mM sodium orthovanadate, 0.2 mM PMSF supplemented with protease inhibitor tablet (Roche Applied Sciences)). Protein concentration was determined by the Bio-Rad Protein Assay reagent (BIO-RAD). Isolated proteins were separated on SDS–PAGE and transferred to PVDF membrane (Millipore). Immunoblotting was carried out with antibodies specific for FOXM1 (Santa Cruz, the rabbit polyclonal antibody against FOXM1 was described previously^28^ and also NOVUS), HSP70 2A4 (a gift from Dr Morimoto), ubiquitin (Santa Cruz), cleaved caspase-3 (Cell signaling), and β-actin (Sigma).

### Luciferase assay

Cells were treated as indicated in the figure legends. The luciferase activity was determined by the Luciferase Assay System (Promega) according to the recommendations of the manufacturer.

### Preparation of FOXM1-DBD recombinant protein

The GST-FOXM1-DBD (221–365) plasmid was a kind gift of Dr Pradip Raychaudhuri. BL21-AI^TM^ *Escherichia coli* cells were transformed with the construct and grown at 37 °C until the optical density reached 0.8. At this point the temperature was decreased to 18 °C, and the expression of GST-FOXM1-DBD was induced by the addition of 1 mM isopropyl-β-d-thiogalactoside, 0.2% arabinose and 2% ethanol for 16 h. Bacteria cells were lysed in PBS by sonication. GST-FOXM1-DBD was purified by incubation with glutathione-Sepharose beads (GE Healthcare) for overnight at 4 °C. The fusion protein was eluted from the beads with 10 mM glutathione elution buffer at room temperature. Glutathione was removed by overnight dialysis. Purity was checked by SDS/PAGE followed by Coomassie staining.

### EMSA

Binding of FOXM1 to dsDNA consensus oligonucleotide (forward strand: 5′-/56-FAM//iSpC3/AAA CAA ACA AAC AAT C -3′) was detected by EMSA. 2 µM of protein and 100 nM dsDNS oligonucleotide were mixed in a 10 µL reaction volume, incubated at room temperature for 1 h. The protein-DNA complex was resolved on a 6% non-denaturing gel run at 4 °C for 45 min at 90 V. The binding buffer consists of 20 mM Tris (pH 8.0), 50 mM KCl, 5 mM MgCl_2_ and 1 mM DTT. For displacement experiments, 100 µM of honokiol was added to the reaction mixture.

### NMR

Saturation transfer difference experiments were performed on Bruker 800 mHz Avance spectrometer equipped with a cryogenic probe. The saturation was achieved with a train of 50 ms Gaussian-shaped pulses applied at field strength of 100 Hz in the methyl region at −1 ppm. The duration of the saturation pulse was 1 s. All experiments were performed with 150 ng recombinant FOXM1 (Abnova) in a phosphate buffered saline (pH 7.6) solution, with drugs dissolved in DMSO and added to a final concentration of 2 mM, and carried out at room temperature. The final volume of DMSO was 10 µL in a total sample volume of 200 µL.

### ChIP

ChIP was performed as described in refs.^4,29^.

### Total RNA extraction and quantitative real-time PCR

To extract total RNA cells were collected by TRIzol reagent (Invitrogen). complementary DNA (cDNA) was synthesized using the High Capacity cDNA Reverse Transcription Kit (Applied Biosystems). Quantitative real time PCR was run using the ABI 7900 HT (Applied Biosystems) machine with primers, as described in refs. ^5,30^.

### Adenoviral transduction

U2OS cells were transduced with control or FOXM1-expressing adenoviral particles for 24 h and treated, as described in the figure legend. Adenovirus expressing LacZ or FOXM1 was a gift from Dr Pradip Raychaudhuri (UIC).

### Statistical analysis

Statistical analysis was performed using one-way ANOVA followed by Tukey’s multiple comparison post test or unpaired *t* test. *P* values of < 0.05 were considered to be statistically significant.
